# Efficacy of self-designed intraoral appliances in prevention of cheek, lip and tongue bite after local anesthesia administration in pediatric patients

**DOI:** 10.4317/jced.55477

**Published:** 2019-04-01

**Authors:** Wala A. Alghamidi, Sondos B. Alghamdi, Jawaher-Ahmad Assiri, Abeer A. Almathami, Zuhair-Motlak Alkahtani, Rafi A. Togoo

**Affiliations:** 1 BDS, Department of Pediatric Dentistry #x00026; Orthodontic Sciences , King Khalid University College of Dentistry, Abha, Saudi Arabia; 2 BDS, Teaching Assistant Department of Pediatric Dentistry #x00026; Orthodontic Sciences , King Khalid University College of Dentistry, Abha, Saudi Arabia; 3 BDS, Department of Restorative Dentistry, King Khalid University, Abha, Saudi Arabia; 4 DPD (Doctor of Pediatric Dentistry), Assistant Professor, Department of Pediatric Dentistry #x00026; Orthodontic Sciences , King Khalid University College of Dentistry, Abha, Saudi Arabia; 5 MDS, Professor, Department of Pediatric Dentistry #x00026; Orthodontic Sciences , King Khalid University College of Dentistry, Abha, Saudi Arabia

## Abstract

**Background:**

The occurrence of self-inflicted soft tissue injuries following administration of local anesthesia in pediatric patients who have received dental treatment has been reported. Aim: To evaluate the attitudes and knowledge regarding cheek, lip, and tongue bite post administration of local anesthesia among dental practitioners in Saudi Arabia. Additionally, the efficacies of three types of intraoral appliances on the prevention of self-harm were evaluated in pediatric patients.

**Material and Methods:**

A total of 301 practitioners were provided with a questionnaire consisting of 9 items. In addition, three types of intraoral appliances made of polyethylene terephthalate were designed as follows: design 1 (consisted of an anterior extension with numerous perforations); design 2 (had a buccal flap extension); and design 3 (comprised of serrated borders). The appliances were placed in the oral cavities of 45 children (age, 3–15 years) immediately after the completion of the dental procedure. The patient was asked to retain the appliance for 3 h. After 24 h, both parents and children were required to respond to a checklist to evaluate the effectiveness the appliances.

**Results:**

Almost half of the dental practitioners had never encountered self-inflicted soft tissue injury in children after local anesthesia administration. About 60% of the dentists were of the opinion that provision of adequate instructions after treatment could prevent the occurrence of lip, cheek, and tongue biting. Furthermore, among the three appliances used, design 1 was most well accepted.

**Conclusions:**

Intraoral appliances used in this study may be considered for use to prevent self-inflicted soft tissue trauma in children following administration of local anesthesia.

** Key words:**Cheek biting, lip biting, intraoral appliance, local anesthesia.

## Introduction

Self-inflicted injuries such as lip and cheek biting are known to be potential complications of local anesthesia following dental treatment, especially in pediatric patients (1-3). Altered sensations or numbness in the lips, cheeks, and tongue last for a few hours post-treatment, and may lead to self-harm in children (2). Recently, an α-antagonist called phentolamine mesylate was shown to reduce the duration of soft tissue associated anesthesia and the incidence of self-injury after a dental procedure (4,5). Similarly, submucosal injection of hydralazine HCl was demonstrated to be safe and effective for the reduction of the duration of local anesthetic-induced anesthesia and its associated problems in 50 patients who received inferior alveolar nerve block (6).

The fabrication of intraoral appliances for prevention of self-inflicted soft tissue injury has been reported in special needs patients (7-9) and in those with habitual biting of the oral mucosa (10,11). Polyethylene terephthalate has been used in the fields of medicine (12,13) and dentistry, particularly orthodontics (14). This material has been used for the fabrication of orthodontic aligners, mouth guards, and splints (15,16).

In the present study, we aimed to evaluate the opinions of dental practitioners in Saudi Arabia, regarding cheek, lip, and tongue bite post administration of local anesthesia. In addition, the efficacies of three types of intraoral appliances in the prevention cheek, lip, and tongue bite were evaluated. 

## Material and Methods

This study was approved by the Ethical Clearance Committee at King Khalid University, Abha, Saudi Arabia. Informed consent was obtained from all participants or their guardians prior to enrollment.

A survey questionnaire designed in English and Arabic was provided to 301 dental practitioners in the cities of Abha and Khamis Mushayat, Saudia Arabia ( Table 1, Table 1 continue). The questionnaire consisted of 9 items pertaining to the occurrence of cheek, lip, and tongue bite after local anesthesia administration in children.

**Table 1:**
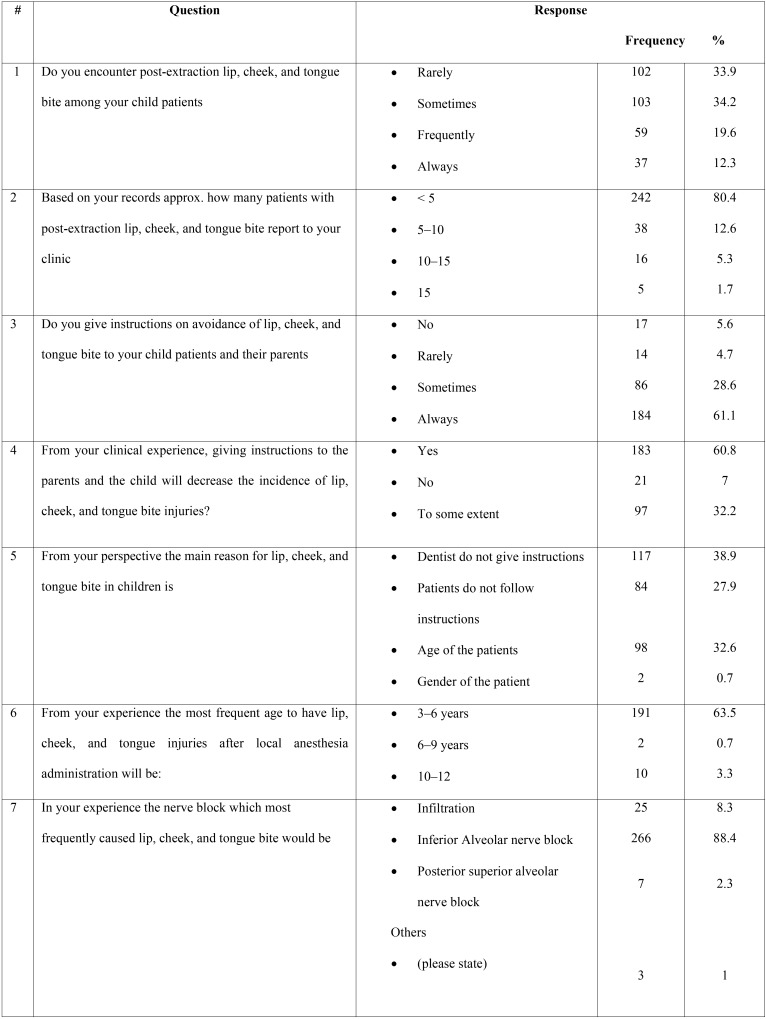
Responses of the dental practitioners to the questionnaire.

**Table 1 continue:**
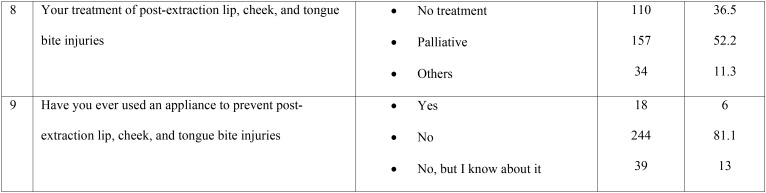
Responses of the dental practitioners to the questionnaire.

**Figure 1:**
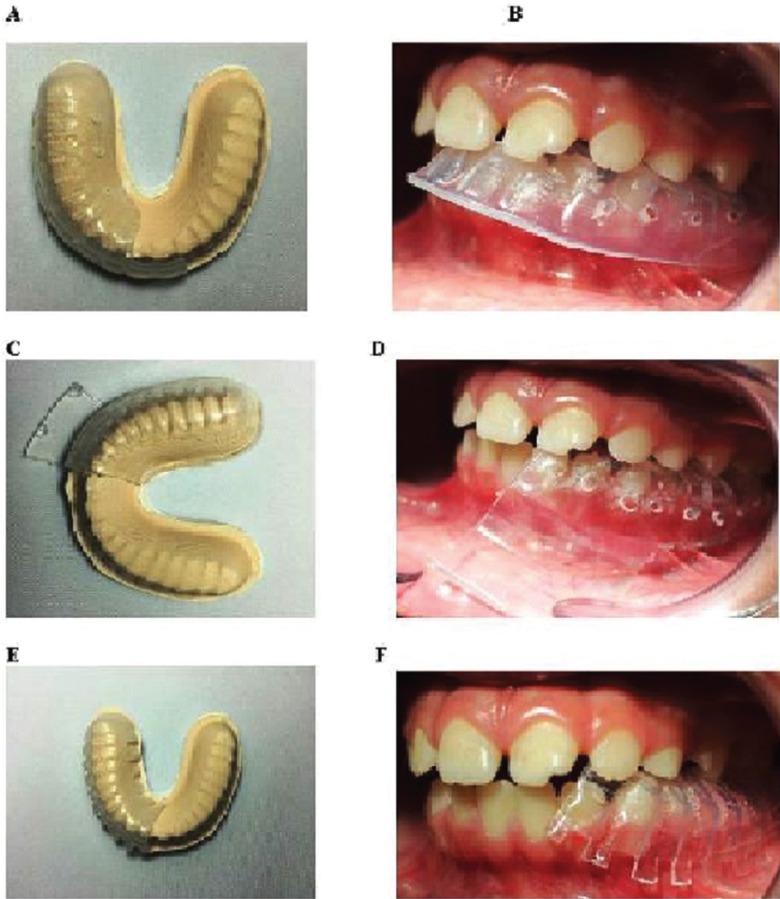
Photomicrographs showing the three types of appliances on the dental cast (A, C, and E) and in the oral cavity (B, D, and F). Design 1 (A, B) consisted of an anterior extension with numerous perforations, design 2 (C, D) had a buccal flap extension, and design 3 (E, F) comprised of serrated borders.

In addition, three types of intraoral appliances made of polyethylene terephthalate, a transparent and flexible material, were fabricated for the children (Fig. 1). The appliances were fabricated in three standard sizes based on the age of the patient: 3-6, 6-9, and 9-12 years. Furthermore, the three designs used in this study were as follows: design 1, which consisted of an anterior extension with numerous perforations (Fig. 1A,B); design 2, which had a buccal flap extension (Fig. 1C,D); and design 3, with serrated borders (Fig. 1E,F). The efficacies of the appliances were tested on 45 children aged between 3-15 years. Informed consent was obtained prior to inserting the appliance. The appliance was placed in the oral cavity immediately after the completion of the dental procedure in patients who received inferior alveolar nerve block using 2 % lidocaine with epinephrine (1:50,000 or 1:100,000). Each patient was asked to retain the appliance in the mouth for 3 h. The patients were recalled after 24 h, and both parents and children were required to respond to a checklist to evaluate the effectiveness and comfort of the appliance ( Table 2).

**Table 2:**
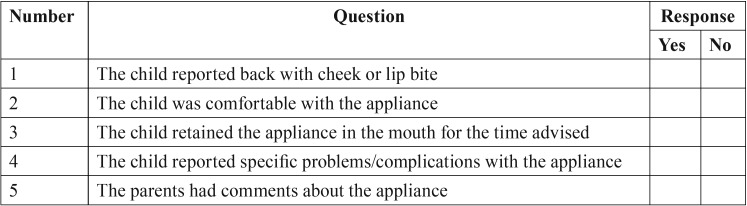
Checklist for the children and parents with regard to the three types of appliances fabricated.

-Statistical analysis

Data was collected using a MS Excel sheet, and analyzed using SPSS, version 20.0 (SPSS Inc, Chicago, IL, USA) to get the frequency distribution of responses. Chi-square test was used to compare frequency of responses from subjects for 3 different designs of appliances.

## Results

A summary of the responses of the 301 dental practitioners to the questionnaire are shown in  Table 1. Ninety-six (31.9%) practitioners reported that they were familiar with post-extraction lip, cheek, and tongue bite; in addition, 102 (33.9%) had rarely encountered such cases in their practice, while 103 of them had occasionally encountered a few of these cases. The majority 191 (63.5%) of the cases reported were in children aged 3 to 6 years, and 89 (32.6%) cases were noted between the ages of 6 to 9 years.

When asked about the reason for the lip, cheek, and tongue bite after the local anesthesia, 117 (38.9%) practitioners blamed the dentists for not providing appropriate instructions after treatment, 98 (32.6%) attributed it to the age of the patient, while 84 (27.9%) believed that refusal to follow instructions provided by the dentists may lead to self-injury in the children.

As seen in  Table 1, more than half of the practitioners (183/301; 60.8%) stated that the provision of proper postoperative instructions could prevent lip, cheek, & tongue bite, whereas less than one-third of them (97; 32.2%) indicated that it may prevent the trauma to some extent only. A total of 184 (61.1%) practitioners reported that they always provided postoperative instructions to their patients, while 86 (28.6) stated that they gave it most of the time; on the other hand, instructions were rarely or never provided by 14 (4.7%) and 17 (5.6%) of the 301 practitioners, respectively. The majority of the practitioners (266; 88.4%) responded that most cases of lip, cheek, and tongue bite were encountered with inferior alveolar nerve block, while 25 (8.3%) and 7 (2.3%) reported that self-inflicted soft tissue was commonly seen after infiltration and posterior superior alveolar nerve block, respectively. Most of the practitioners 157 (52.2%) dealt with the cases in a palliative manner, whereas 110 (36.5%) of them did not provide any treatment to the patients. Furthermore, 244 (81.1%) dental practitioners had never used or read about the use of an appliance that could prevent the occurrence of complications after local anesthesia administration; 39 (13%) had only read about them, and 17 (6%) reported having used such appliances.

**Figure 2:**
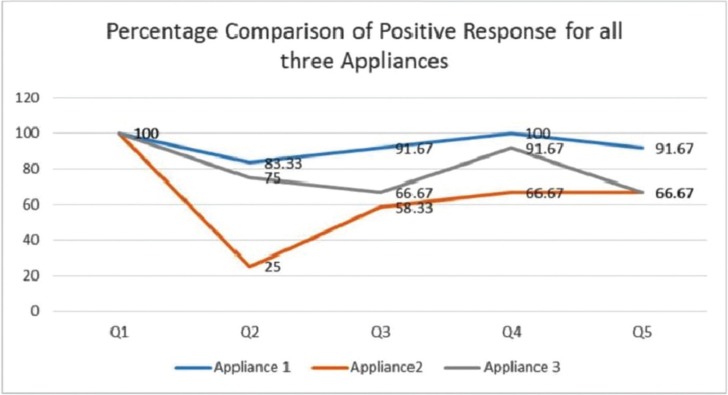
Graph showing the percentage of favorable responses received from the 45 children and their parents to the five questions regarding the three different types of appliances. Design 1 received the most favorable responses among the three.

Out of the three types of intraoral fabricated in this study, design 1 received the most favorable response when compared with designs 2 and 3 based on the checklist created. Design 1 was superior in comfort, and received positive responses from 83.3% of the children. Moreover, 100% of children and 91.67% of parents did not report any complaints with design 1 while 91.7% and 66.7% of children and parents respectively, had no complaints for design 2; 66.67% and 58.33% of children and parents respectively, did not mention any complaints regarding design 3 (Fig. 2).

33.3% and 8.8% of parents and children respectively had some complaints about design 2 while 42.2% and 33.33% of parents and children respectively had few complaints about design 3. All three types of appliances were retained in the mouth for the recommended periods of time advised by the children, except for design 2 (58.3% of the children).

## Discussion

The effects of anesthesia can last for several hours irrespective of the type of local anesthesia used (1,17). Moreover, it has recently been shown that the use of mandibular infiltrations, instead of blocks does not reduce the occurrence of these injuries (18); however, according to Vempaty and Robbins (2017), the use of short acting anesthetics may prevent self-harm in children (2). The most common local anesthetic agents used in dentistry are 2% lidocaine with epinephrine (1:100,000). In the current study, the two agents were administered via inferior alveolar nerve block at a ratio of 1:50,000 or 1:100,000. Nevertheless, approximately 90% of the dental practitioners in this study identified inferior alveolar nerve block as the mode of anesthesia that most commonly caused lip, cheek or tongue biting in children.

More than half the number of dental practitioners in the present study had rarely or infrequently encountered lip, cheek or tongue biting among pediatric patients who had received local anesthesia for dental treatment. In addition, the majority of the children presenting with these conditions belonged to the 3-6 age group. These findings are similar to those reported by College *et al.* (2000), wherein 13% of 320 children aged 2 to 18 experienced soft tissue trauma after local anesthesia, with the highest frequency observed in children below 4 years of age (19). In another study, 14 (4%) out of 349 children (age range, 2-18) presented with lip biting after local anesthesia (3).

Interestingly, with regard to the question about the provision of instructions to the patients, 61% of the dentists responded that they always provided the required instructions to the patients after treatment, whereas the proportion of practitioners who rarely or never gave instructions was about 10%. Similarly, nearly 61% of the dentists believed that the provision of instructions to the patients could decrease the occurrence lip, cheek, and tongue biting after local anesthesia, while 7% did not endorse the opinion. Approximately 40% of the practitioners felt that lack of provision of adequate instructions accounted for the occurrence of these self-inflicted injuries, while nearly 30% blamed the patients for not following the instructions. It has been suggested that creating awareness of the time of action of the local anesthetic agents, and the possibility of self-injury may help prevent or reduce the occurrence of lip, cheek, and tongue biting among children (1,2).

Most self-inflicted injuries following local anesthesia are minor and resolve on their own, or may require palliative care such as analgesics or chlorhexidine rinses. More than half of the dentists in the current study have provided palliative care for the patients when required. Ram *et al.* (2010) reported that the licking of a popsicle after dental treatment can reduce the incidence of soft tissue trauma in children, while Vempaty and Robbins (2017) suggested that avoiding food during this time might prevent the occurrence of lip, cheek, and tongue biting (2,20). However, the use of intraoral appliances to prevent the occurrence of soft tissue injury following local anesthesia has not been reported so far.

Intraoral appliances have been used for the prevention of self-inflicted soft tissue injuries in children with developmental and psychological problems. Removable shields made of soft silicone, soft polyvinyl splints, mouth guards, and cheek plumpers have been used to prevent tongue, lip, and cheek biting (11,21-23). In the present study, three types of intraoral appliances made of polyethylene terephthalate were fabricated and tested on 45 children aged between 3-15 years. Polyethylene terephthalate glycol is a clear, light, resistant, and elastic material that has been used for orthodontic purposes (24,25). Based on the checklist questionnaire provided to the children and parents, design 1 appeared to be well accepted. As seen in figure 1, the appliance with design 1 consisted of an anterior extension with several perforations, which provided good retention in the oral cavity. On the other hand, design 2 consisted of a buccal flap extension leading to poor retention because of the pushing action of the flap against the buccal mucosa. Similarly, the serrated borders in design 3 allowed for the escape of saliva through the gaps, thereby resulting in poor retention of the appliance in the oral cavity.

## Conclusions

The findings of this study provide information about the attitudes and awareness of dental practitioners with regard to lip, cheek, and tongue biting in children who receive local anesthesia for dental treatment. The provision of adequate instructions to the patient is vital. The parent must monitor the child for a few hours after the treatment. Furthermore, among the three appliances fabricated in the current study to prevent self-injury in children, design 1 was most accepted and may be considered for use as a conservative approach for the management of self-inflicted soft tissue trauma in children following local anesthesia.
